# Insulin, Insulin Everywhere: A Rare Case Report of Rabson-Mendenhall Syndrome

**DOI:** 10.7759/cureus.13126

**Published:** 2021-02-04

**Authors:** Siddharth Gosavi, Samarth Sangamesh, Amogh Ananda Rao, Suman Patel, Vishwakarma C Hodigere

**Affiliations:** 1 Internal Medicine, Jagadguru Jayadeva Murugarajendra (JJM) Medical College, Davangere, IND

**Keywords:** insulin receptor, diabetes mellitus, rare genetic diseases, insulin resistance, rabson-mendenhall syndrome (rms)

## Abstract

Rabson-Mendenhall syndrome (RMS) is a rare autosomal recessive disorder characterized by severe insulin resistance, a condition in which the body’s tissues and organs do not respond appropriately to the hormone insulin. Insulin resistance impairs blood sugar regulation and ultimately leads to diabetes mellitus. A 19-year-old male presented with joint pain, blurring of vision, and generalized weakness. Investigations revealed hyperglycemia (random blood sugar (RBS) > 625 mg/dL, glycosylated hemoglobin (HbA1c) 18%), as well as sugars, protein, and ketone bodies in urine routine examination. An ultrasound of the abdomen was normal. Cardiac status was normal. Based on the clinical features, particularly the head to toe examination, skin changes, and the onset of type 2 diabetes mellitus, RMS syndrome was considered. The joint pain was alleviated with intravenous tramadol. Actrapid®, a fast-acting insulin, was given to control sugar levels, along with metformin. Vitamin B12 and pregabalin were also supplemented. A dermatological cream containing ammonium chloride, calcium lactate, glycerin, potassium chloride, sodium dihydrogen phosphate, and urea was given for skincare. It is an extremely rare disease with a frequency of fewer than one million people worldwide. Most patients survive only up to 15 years of age, although some can live into their third decade of life. Hepatic gluconeogenesis and fatty acid oxidation are affected, leading to ketoacidosis. The progression is much faster in RMS. In RMS, the genetic defect affects the insulin receptor (INSR) gene transcription with non-sense mutations and causes splicing defects. This results in premature chain termination and eventually to lower amounts of the insulin receptor messenger ribonucleic acid (mRNA). Ultimately, the number and density of insulin receptors in the plasma membrane are smaller, making the cells resistant to insulin. Herein, we report the case of a 19-year-old patient with RMS who was treated in our hospital, leading to a successful improvement in symptoms and discharge of the patient.

## Introduction

Rabson-Mendenhall syndrome is a genetic syndrome epitomized by severe insulin resistance, where there is an inappropriate response to insulin by the tissues and organs. It is extremely rare, occurring in less than one in a million people, and is an autosomal recessive disorder. Insulin resistance leads to impairment of blood sugar levels in the body and eventually leads to diabetes mellitus. Severe insulin resistance in people with RMS affects the development of many parts of the body. Affected individuals present with small stature, lack of subcutaneous fat, atrophy of muscles, dental abnormalities, hirsutism, multiple cysts in the ovaries, and enlargement of the nipples and genitalia. The skin in body folds and creases becomes thick, dark, and velvety, called acanthosis nigricans [1].

Patients with RMS also present with prominent, widely spaced eyes, a broad nose, and large low set ears. Other inherited insulin resistance syndromes include Donohue syndrome and type A insulin resistance syndrome. RMS is intermediate in severity amongst them. People with RMS develop signs and symptoms early in life and live into their second or third decade. Death usually results from complications related to diabetes mellitus, such as diabetic ketoacidosis [2].

## Case presentation

A 19-year-old male presented to Chigateri General Hospital attached to the Jagadguru Jayadeva Murugarajendra (JJM) Medical College, Davangere, India, in April 2020 with complaints of joint pain for one year and generalized weakness for 20 days. He also had progressive and painless blurring of vision for the last two months, more so in the right than the left eye. There was no photophobia, lachrymation, or trauma history. He was diagnosed with diabetes mellitus six years earlier and has been managed with insulin, metformin, and pioglitazone. 

His parents had a third-degree consanguineous marriage; the sibling is unaffected. He has a maternal cousin with similar skin lesions. His mother did not avail herself of antenatal care. She underwent a domicile delivery to give birth to the patient. Immunization history is inconsistent. 

There was no history of palpitation, swelling of lower limbs, syncope, reduced urine output, no history of hypertension, congenital heart disease, kidney disease, hypothyroidism, or any other illness.

The patient was poorly built and undernourished. His vital parameters were normal; there were no signs of pallor, icterus, cyanosis, clubbing, lymphadenopathy, or edema. His body mass index was 15.4 kg/m^2^. The middle upper arm circumference was 16.5 cm, and head circumference was 51.5 cm.

**Figure 1:**
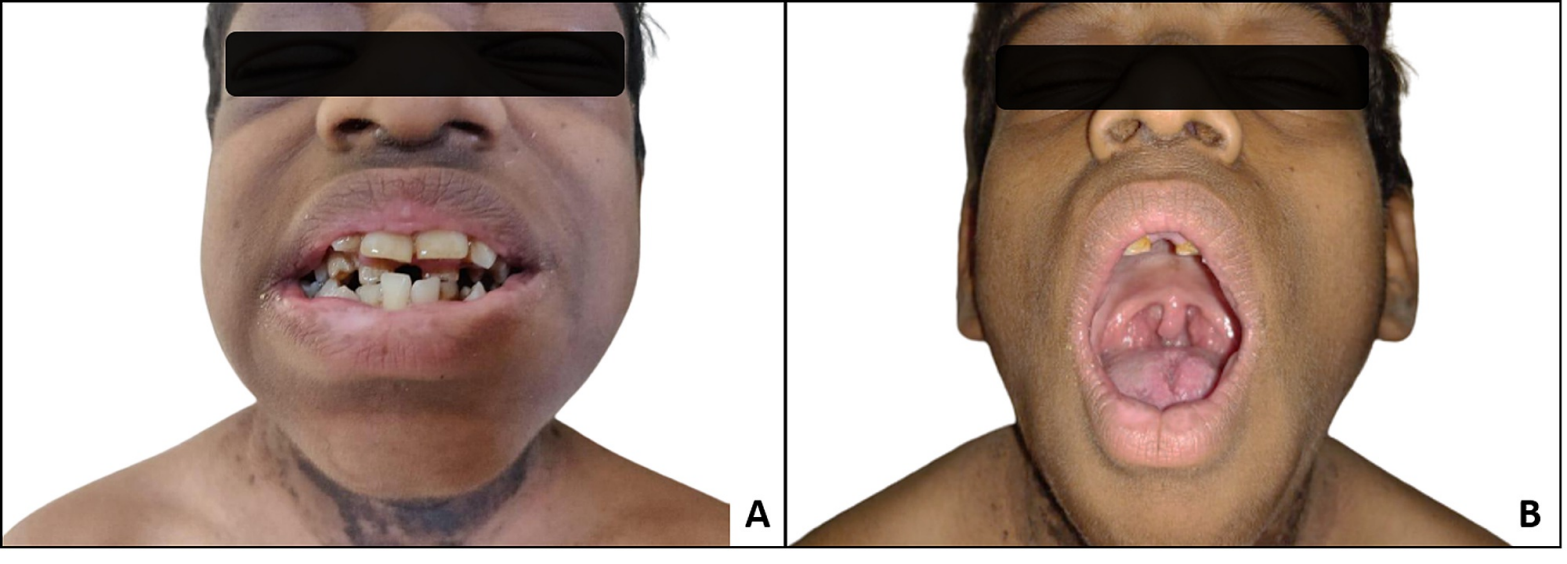
A: coarse facies with malocclusion of teeth with hyperdontia; B: fissured tongue and enlarged tonsils

**Figure 2:**
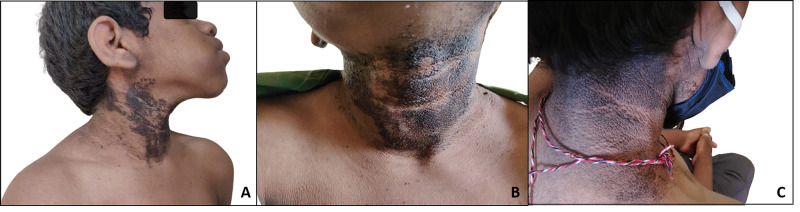
A: prognathism; A-C: dermatitis neglecta with underlying acanthosis nigricans

**Figure 3:**
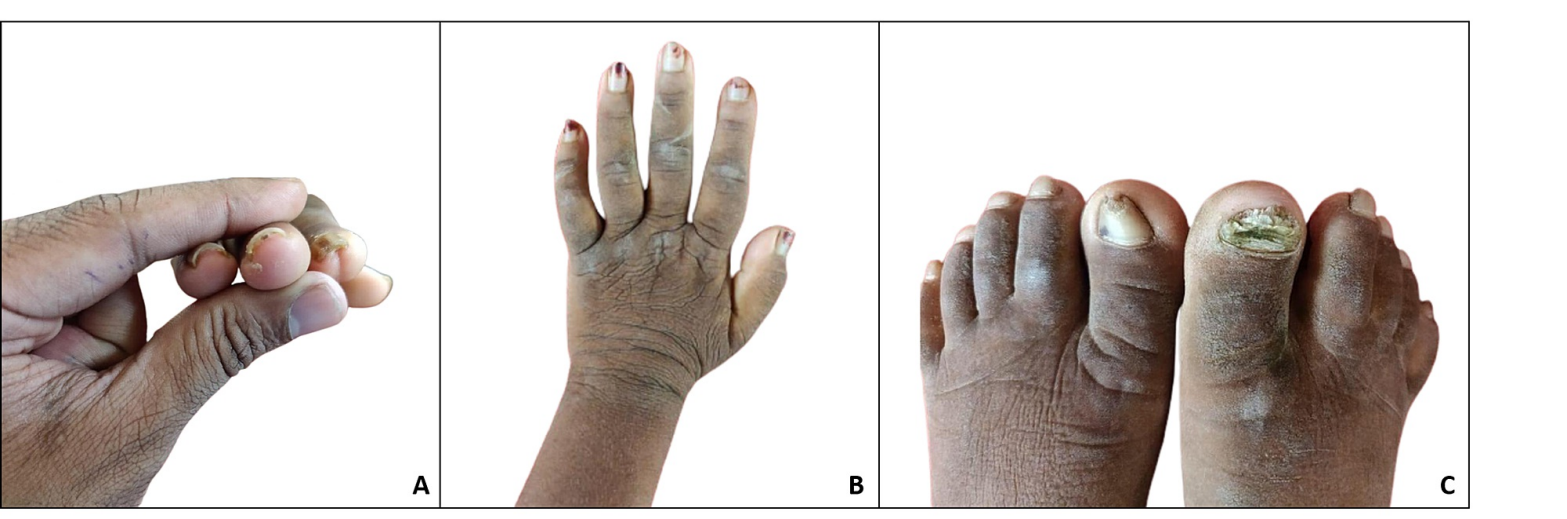
A: subungual hyperkeratosis; B: acanthosis nigricans on the dorsum of the hand extending to the forearm; C: onychodystrophy of the nail of the right great toe with a pincer nail of the left great toe

He had large prominent eyes with coarse facies and malocclusion of the teeth with hyperdontia (Figure 1A). Dermatitis neglecta with acanthosis nigricans was present on all sides of the neck, including the posterior aspect (Figure 2A-C). A fissured tongue was observed, along with enlarged tonsils (Figure 1B). Subungual hyperkeratosis was also present in his fingernails (Figure 3A). Acanthosis nigricans was also present on the dorsum of the hand extending to the forearm (Figure 3B). His toenails showed onychodystrophy and pincer nail (Figure 3C. His liver was clinically enlarged, and the examination of all other systems was unremarkable.

The various blood investigations and imaging studies are presented in Table 1.

**Table 1:** Blood Investigations and Imaging Studies A/G ratio: albumin/globulin ratio; Echo: echocardiogram; HbA1c - glycosylated hemoglobin; RBCs: red blood cells; RBS: random blood sugar; TSH: thyroid-stimulating hormone; 2D: two-dimensional; USG: ultrasonography; WBCs: white blood cells

GLYCEMIC STATUS		LIVER AND KIDNEY FUNCTION	
RBS	> 625 mg/dL	Serum urea	42 mg/dL
HbA1C	18%	Serum creatinine	0.5 mg/dL
BLOOD COUNT		Total bilirubin	0.3 mg/dL
Haemoglobin	13.9 g/dL	Total protein	6.2 gm/dL
WBC Count	8,200 cells/ml	A/G ratio	1
Platelets	2.73 lakh/cumm	Serum electrolytes	Normal
THYROID PROFILE		URINE ANALYSIS	
T3	0.72 ng/ml	Physical Examination	Turbid
T4	7.74 mcg/dL	pH	5.5
TSH	1.39 microIU/ml	Sugar	4+
RADIOLOGY		Protein	3+
2D Echo	Normal	Ketone bodies	++
USG Abdomen		RBCs	Nil
Chest x-ray		Nitrite	-

Retinoscopy revealed severe proliferative diabetic retinopathy in the right eye and severe non-proliferative diabetic retinopathy in the left eye. However, a limitation of this report is that genetic studies could not be performed due to the financial constraints of the patient's family. Donohue syndrome was naturally ruled out as affected individuals hardly survive their first two years of life. Clinical pointers towards lipodystrophy were absent and the parents were unaffected, ruling out autosomal dominant inheritance. Therefore, a diagnosis of RMS was made.

The excruciating joint pain was alleviated with intravenous tramadol, 50 mg per day. Insulin Actrapid was subcutaneously administered based on a regular insulin sliding scale protocol to control sugar levels, along with metformin, 500 milligrams per day. Vitamin B_12_, 100 micrograms administered intramuscularly once per day, and pregabalin, 75 mg once daily were also supplemented. A dermatological cream containing ammonium chloride, calcium lactate, glycerin, potassium chloride, sodium dihydrogen phosphate, and urea was given for skincare. The patient responded well to the treatment and was discharged after a one week stay. 

## Discussion

The suboptimal glucose response to a given concentration of insulin is called insulin resistance [3]. Obesity, excess cortisol, growth hormone, catecholamines, glucagon, and genetic defects in insulin signaling are major etiological factors contributing to insulin resistance. Notably, in genetic syndromes, patients have extreme insulin resistance. Initially, the patient will have normoglycemia with a simultaneous rise in insulin levels. The patient presents with a higher postprandial sugar reading before the onset of well-sustained hyperglycemia. This is due to patients with insulin receptor (INSR) defects having an improper hepatic insulin clearance or hepatic steatosis. A minority of patients present with hypoglycemia due to activated autoantibodies against the INSR [4-7].

In RMS, a mutation of the INSR gene leads to a severe compromise of the INSR function. An interaction between insulin and IGF-1 receptors leads to a conspicuous delay in linear growth and failure to thrive [4]. Fasting insulin is one of the standard tests employed to diagnose RMS. The cut-off point to characterize hyperinsulinemia is 15.7 mcU/ml. Hyperinsulinemia can be used as an indicator to further evaluate the patient for the presence of INSR mutations, circulating anti-INSR antibodies [8].

**Figure 4:**
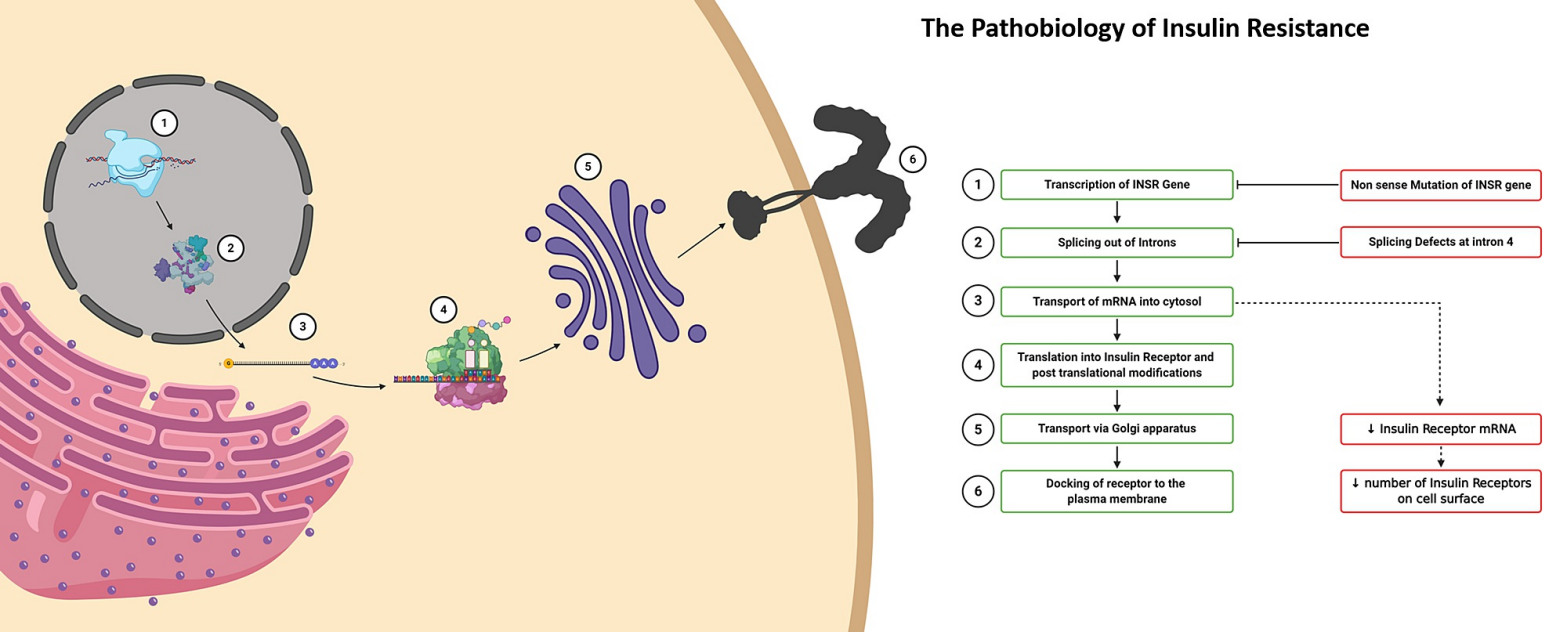
The pathobiology of insulin resistance in Rabson Mendenhall syndrome (RMS) Non-sense mutations in the transcription of the insulin receptor (INSR) gene and splicing defects cause an overall decrease in the messenger ribonucleic acid (mRNA) coding for the INSR, eventually leading to a decreased density of INSRs on the plasma membrane [9-10].

In RMS, the genetic defect affects the transcription of the INSR gene with non-sense mutations and causes splicing defects. This results in premature chain termination and eventually to lower amounts of INSR mRNA. Ultimately, the number and density of INSRs in the plasma membrane are smaller, making the cells resistant to insulin (Figure 4) [9-10].

There is no specific treatment for patients with RMS and thus treatment is ordinarily supportive. The treatment plan's primary objective is to achieve glycemic control, delay, and alleviate the symptoms of complications. The role of insulin sensitizers and biguanides are debatable. Nonetheless, recombinant human (rh) insulin-like growth factor I (IGF-I) and recombinant methionyl human leptin (r-metHuLeptin) show promise in treating ketoacidosis and improvement of insulin tolerance, respectively. Diabetic ketoacidosis and fasting hypoglycemia are the major causes of mortality in RMS [2].

## Conclusions

Genetic counseling is an extremely crucial factor and plays an important role in explaining the prognosis of genetic diseases and helping the patients and their families to develop awareness and understanding about the condition. The symptoms of genetic diseases can only be controlled. As was observed in this case, glycaemic control was achieved but there is no permanent cure for RMS. A larger focus is warranted on genetic diseases and their diagnosis, especially in a developing country like India where facilities to diagnose genetic diseases are stillevolving and are at a nascent stage.
